# Robust signatures detection of Majorana fermions in superconducting iron chains

**DOI:** 10.1038/srep36600

**Published:** 2016-11-18

**Authors:** Hua-Jun Chen, Xian-Wen Fang, Chang-Zhao Chen, Yang Li, Xu-Dong Tang

**Affiliations:** 1 School of Science, Anhui University of Science and Technology, Huainan Anhui, 232001, China

## Abstract

We theoretically propose an optical means to detect Majorana fermions in superconducting iron (Fe) chains with a hybrid quantum dot-nanomechanical resonator system driven by two-tone fields, which is very different from the current tunneling spectroscopy experiments with electrical means. Based on the scheme, the phenomenon of Majorana modes induced transparency is demonstrated and a straightforward method to determine the quantum dot-Majorana fermions coupling strength is also presented. We further investigate the role of the nanomechanical resonator, and the resonator behaving as a phonon cavity enhances the exciton resonance spectrum, which is robust for detecting of Majorana fermions. The coherent optical spectrum affords a potential supplement to detecte Majorana fermions and supports Majorana fermions-based topological quantum computation in superconducting iron chains.

Majorana fermions (MFs) are real solutions of the Dirac equation and which are their own antiparticles *γ* = *γ*^†^. Although proposed originally as a model for neutrinos, MFs have recently been predicted to occur as quasi-particle bound states in engineered condensed matter systems^1^. This exotic particle obeys non-Abelian statistics, which is one of important factors to realize subsequent potential applications in decoherence-free quantum computation^2^ and quantum information processing^3^. Over the recent few years, the possibility for hosting MFs in exotic solid state systems focused on topological superconductors^1^,^2^,^3^,^4^. Currently, various realistic platforms including topological insulators^5^,^6^, semiconductor nanowires (SNWs)^7^,^8^, and atomic chains^9^,^10^,^11^,^12^,^13^ have been proposed to support Majorana states based on the superconducting proximity effect. Although various schemes have been presented, observing the unique Majorana signatures experimentally is still a challenging task to conquer.

MFs are their own antiparticles, they therefore are predicted to appear in tunneling spectroscopy experiments, and MFs manifest themselves as characteristic zero-bias peaks (ZBPs)^14^,^15^. Theoretical predictions of ZBPs have been observed experimentally in SNWs and interpreted as the signatures of MFs^16^,^17^,^18^,^19^,^20^. Nadj-Perge *et al*.^21^ recently designed a chain of magnetic Fe atoms deposited on the surface of an s-wave superconducting Pb with strong spin-orbit interactions, and reported the striking observation of a ZBP at the end of the atomic chains with STM, which provides evidence for Majorana zero modes. However, these above experimental results can not serve as definitive evidences to prove the existence of MFs in condensed matter systems, and it is also a major challenge in these experiments to uniquely distinguish Majorana from conventional fermionic subgap states. The first reason is that the zero-bias conductance peaks can also appear in terms of the other mechanisms^22^,^23^, such as the zero-bias anomaly due to Kondo resonance^20^,^24^ and the disorder or band bending in the SNW^25^. The second one is that Andreev bound states in a magnetic field can also exhibit a zero-energy crossing as a function of exchange interaction or Zeeman energy^26^,^27^, which therefore gives rise to similar conductance features. The experimental evidences for Majorana bound states largely relies on measurements of the tunneling conductance at present, and the observation of Majorana signature based on electrical methods still remains a subject of debate. Identifying MFs only through tunnel spectroscopy is somewhat problematic. To obtain definitive MFs signatures, alternative setups or proposals for the detection of MFs are necessary. Very recently, measuring the splitting of near-zero-energy Majorana modes with the Coulomb blockade spectroscopy is demonstrated experimentally^28^, which is the first systematic measurement of the ground-state degeneracy associated with Majorana zero modes and is a milestone event indicating one step closer to topological quantum computation.

In the present work, we propose an alternative all-optical measurement scheme to detect the existence of MFs in iron chains on the superconducting Pb surface^21^ via a coupled hybrid quantum dot-nanomechanical resonator (QD-NR) system^29^,^30^ driven by two-tone fields^31^. In the optical scheme, there is no direct contact between MFs and the hybrid QD-NR system, which can effectively avoid introducing other signals to disturb the detection of MFs. The QD is considered as a two-level system rather than a single resonant level with spin-singlet state in electrical detection scheme^32^,^33^,^34^,^35^,^36^,^37^, and once MFs appear in the end of iron chains and couple to QD, Majorana signatures can be carried out via the coherent optical spectra. The signals in the coherent optical spectra indicating a possible signature of MFs are another potential evidence in the iron chains. The optical scheme will provide another method for probing MFs, which is very different from the ZBP in the tunneling experiments^16^,^17^,^18^,^19^,^20^,^21^. In order to investigate the role of the NR in the hybrid system, we further introduce the exciton resonance spectrum to probe MFs. The results shows that the vibration of the NR acting as a phonon cavity will enhance the exciton resonance spectrum significantly and make MFs more sensitive to be detectable.

## Results

### The Model of The System

**Figure 1:**
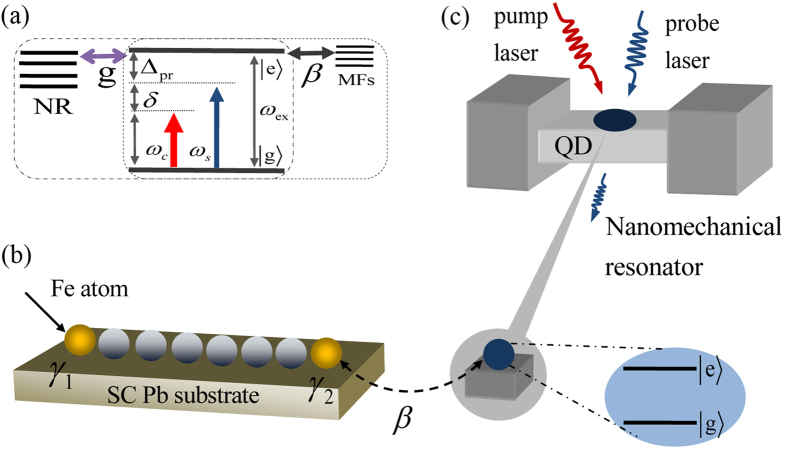
Sketch of the proposed setup for all-optical detection of MFs. (**a**) The energy-level diagram of a QD coupled to MFs and NR, which includes two kinds coupling, i.e. the QD-MF coupling (the dotted frame) and the QD-NR coupling (the dashed frame). (**b**) The iron chains on the superconducting Pb surface, and a pair of MFs appear in the ends of the iron chains. (**c**) The nearby MF is coupled to a QD embedded in a NR driven by two-tone fields.

Figure 1(b) shows the schematic setup that will be studied in this work, where iron (Fe) chains on the superconducting Pb(110) surface^21^, and we employ a two-level QD driven by two-tone fields to detect MFs. The Fe chain is ferromagnetically ordered^21^ with a large magnetic moment, which takes the role of the magnetic field in the nanowire experiments^16^. Different from the proposal of Mourik *et al*.^16^, this “magnetic field” is mostly localized on the Fe chain, with small leakage outside, and superconductivity is not destroyed along the chain. In this setup, the energy scale of the exchange coupling of the Fe atoms is typically much larger than that of the Rashba spin-orbit coupling and the superconducting pairing. Figure 1(c) displays that a QD is implanted in the NR to form a coupled hybrid QD-NR system.

There are two hybrid system in the model, i.e. the hybrid Fe chains/Pb superconducting system and QD/NR system. The Fe ferromagnetic chains grow out of a central island along atomic rows on the crystalline Pb surface, the strong spin-orbit coupling in Pb leads to an effective p-wave component in the induced superconductivity in the Fe chain, and zero-bias peaks as Majorana signature at the end of the chains are discovered with scanning tunneling microscope experiment^21^. While in the QD/NR system, the QD is embedded in the NR, which is a intermediary to detect MFs driven by two-tone fields. Since the model includes complex many body system, it is necessary to explain the collective modes system. In the theoretical model, the hybrid QD-NR system is not embedded in the Pb superconducting substrate, so the coupling effect of the NR system to the Pb superconducting state is not considered in our model. The whole model can be simplify as a three modes coupling system, i.e. NR-QD-MF three coupling modes system where the QD is a intermediary. The whole system includes two kinds of couplings which are QD-MF coupling and QD-NR coupling as shown in Fig. 1(a), and we will discuss the two kinds of coupling in detail in the following text. The coexistence and competition of the two kinds of coupling depend on the parameter conditions.

### The Hamiltonian of QD-MF Coupling and QD-NR Coupling

In the hybrid QD-NR system, QD is modeled as a two-level system consisting of the ground state 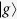 and the single exciton state 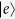 at low temperatures^38^,^39^, and the Hamiltonian of the QD can be described as *H*_*QD*_ = *ħω*_*e*_*S*^*z*^ with the exciton frequency *ω*_*e*_, where *S*^*z*^ and *S*^±^ are the pseudospin operator with commutation relations [*S*^*z*^, *S*^±^] = ±*S*^±^ and [*S*^+^, *S*^−^] = 2*S*^*z*^. For the NR, the thickness of the beam is much smaller than its width, the lowest-energy resonance corresponds to the fundamental flexural mode that will constitute the resonator mode^29^ which can be characterized by a quantum harmonic oscillator with Hamiltonian *H*_*NR*_ = *ħω*_*n*_(*b*^+^*b* + 1/2) with the resonator frequency *ω*_*n*_ (*b* is the annihilation operator of the resonator mode). The flexion induces extensions and compressions in the structure^40^, and the longitudinal strain will modify the energy of the electronic states of QD through deformation potential coupling. The coupling between the resonator mode and QD is described by *H*_*int*_ = *ħω*_*n*_*gS*^*z*^(*b*^+^ + *b*) with the coupling strength *g*^29^. The vibrational direction of the fundamental flexural mode is perpendicular to the direction of the resonator surface. When the QD is embedded in the NR, the QD will also vibrate slightly accompanying with the vibration of NR in the perpendicular direction. In this situation, the distance between the QD and MF almostly does not change, and the MF-dot coupling does not affect the resonant modes of the resonator. Therefore, the coupling of MF-NR can be neglected safely. Then the Hamiltonian of the coupled hybrid QD-NR system is


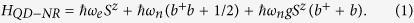


For the QD-MF coupling, as each MF is its own antiparticle, an operator *γ* with *γ*^†^ = *γ* and *γ*^2^ = 1 is introduced to describe MFs. The Hamiltonian of QD couples to the nearby MF *γ*_1_ is^32^,^33^,^34^,^35^,^36^


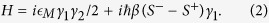


We switch the Majorana representation to the regular fermion one via the exact transformation *γ*_1_ = *f*^†^ + *f (γ*_2_ = *i*(*f*^†^ − *f*)), where *f*^†^ is the fermion creation operator obeying the anti-commutative relation {*f, f*^†^} = 1. Therefore, the Hamiltonian *H* can be rewritten as follows in the rotating wave approximation^41^


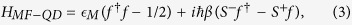


where the first term gives the energy of MF with frequency *ω*_*M*_ and 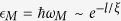 with the iron chains length (*l*) and the Pb superconducting coherent length (*ξ*). If the iron chains length (*l*) is large enough, 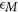 will approach zero. Here, we will discuss the two conditions, i.e., 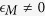 and 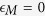, and define the two conditions as coupled MFs (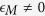) and uncoupled MFs (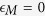), respectively. The second term describes the MF-QD coupling with the coupling strength *β* which is related to the distance of the hybrid QD-NR system and the iron chains. The non-conservation term of energy *iħβ*(*S*^−^*f* − *S*^+^*f*^+^) is neglected, and the numerical calculations (not shown in the following figures) and the results show that the effect of the term is too small to be considered in our theoretical treatment.

When the hybrid QD-NR system is driven by two-tone fields^31^, the Hamiltonian of QD coupled to the two fields is given by^42^ 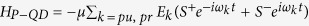, where *μ* is the dipole moment of the exciton, and *E*_*k*_ is the slowly varying envelope of the field, and then the Majorana signature will be carried out via the coherent optical spectrum of the QD. In a rotating frame of the frequency *ω*_*pu*_, we obtain the total Hamiltonian of the system as


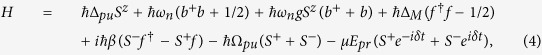


where Δ_*pu*_ = *ω*_*e*_ − *ω*_*pu*_ (*δ* = *ω*_*pr*_ − *ω*_*pu*_, Δ_*M*_ = *ω*_*M*_ − *ω*_*pu*_) is the detuning of the exciton frequency (probe field, the MF frequency) from the pump frequency, respectively. Ω_*pu*_ = *μE*_*pu*_/*ħ* is the Rabi frequency of the pump laser. Actually, we have neglected the regular fermion like normal electrons in the nanowire that interact with the QD in the above discussion. To describe the interaction between the normal electrons and the exciton in QD, a tight binding Hamiltonian of the whole iron chains is introduced^43^.

### The Quantum Langevin Equations

Writing the Heisenberg equations of motion and adding dissipation of the corresponding damping and noise terms, the quantum Langevin equations can be derived as follows,


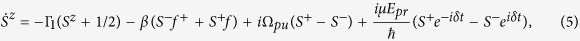



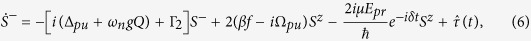



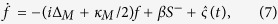



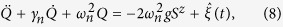


where Γ_1_ (Γ_2_) is the exciton spontaneous emission rate (dephasing rate), *Q* = *b*^+^ + *b* is the position operator, *γ*_*n*_ (*κ*_*M*_) is the decay rate of the NR (MF). 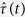 is the *δ*-correlated Langevin noise operator with zero mean and obeys the correlation function 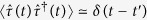. The resonator mode is affected by a Brownian stochastic force with zero mean value 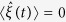 and 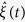 has the correlation function


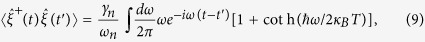


in which *k*_*B*_ (*T*) are the Boltzmann constant (the temperature) of the reservoir of the coupled system. MFs have the same correlation relation as the resonator mode


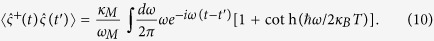


In Eq. (9) and Eq. (10), both the NR and Majorana mode will be affected by a thermal bath of Brownian and non-Markovian processes^44^. In low temperature, the quantum effects of both the Majorana and NR mode are observed only in the case of *ω*_*M*_/*κ*_*M*_ ≫ 1 and *ω*_*n*_/*γ*_*n*_ ≫ 1. Due to the weak coupling to the thermal bath, the Brownian noise operator can be modeled as Markovian processes. In addition, both the QD-MFs coupling and QD-NR mode coupling are stronger than the coupling to the reservoir that influences the two kinds coupling. Owing to the second order approximation^44^, we therefore obtain the form of the reservoir that affects both the NR mode and Majorana mode as in Eq. (9) and Eq. (10).

### The Coherent Optical Spectra

The probe field is much weaker than the pump field, following the standard methods of quantum optics, each Heisenberg operator can be rewritten as the sum of its steady-state mean value and a small fluctuation with zero mean value *O* = *O*_0_ + *δO (O* = *S*^*z*^, *S*^−^, *f, Q*). The steady-state values are governed by the pump power and the small fluctuations by the probe power. Disregarding the probe field, the time derivatives vanish, and the static solutions for the population inversion (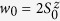) of the exciton obey the following algebraic equation


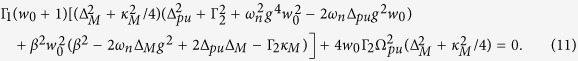


Keeping only the linear terms of the fluctuation operators, we make the ansatz^42^ 〈*δO*〉 = *O*_+_*e*^−*iδt*^ + *O*_−_*e*^*iδt*^. Solving the equation set and working to the lowest order in *E*_*pr*_ but to all orders in *E*_*pu*_, we obtain the linear susceptibility as 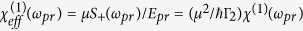, and *χ*^(1)^(*ω*_*pr*_) is given by


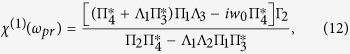


where Σ_1_ = *β*/(*i*Δ_*M*_ + *κ*_*M*_/2 − *iδ*), Σ_2_ = *β*/(−*i*Δ_*M*_ + *κ*_*M*_/2 − *iδ*), 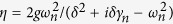, 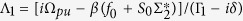, 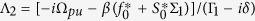, 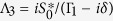, Π_1_ = 2(*βf*_0_ − *i*Ω_*pu*_) − *iω*_*n*_*gS*_0_*η*, Π_2_ = *i*(Δ_*pu*_ − *δ* + *ω*_*n*_*gQ*_0_) + Γ_2_ − *βw*_0_Σ_1_ − Λ_2_Π_1_, Π_3_ = 2(*gf*_0_ − *i*Ω_*pu*_) − *iω*_*n*_*gS*_0_η^*^, Π_4_ = *i*(Δ_*pu*_ + *δ* + *ω*_*n*_*gQ*_0_) + Γ_2_ − *βw*_0_Σ_2_ − Λ_3_Π_3_. The imaginary and real parts of *χ*^(1)^(*ω*_*pr*_) indicate absorption and dispersion, respectively. In addition, the average population of the exciton states can obtain as


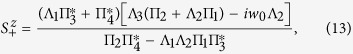


which is benefited for the readout of the exciton states.

We introduce the parameters of the realistic hybrid systems of the coupled QD-NR system^29^ and the iron chains on the superconducting Pb surface^21^. For an InAs QD in the coupled QD-NR system, the parameters are^29^: the exciton relaxation rate (the exciton dephasing rate Γ_2_) Γ_1_ = 0.3 GHz (Γ_2_ = 0.15 GHz). The physical parameters of GaAs NR are (*ω*_*n*_, *M, Q*_*f*_) = (1.2 GHz, 5.3 × 10^−18^ kg, 3 × 10^4^), where *ω*_*n*_, *M*, and *Q*_*f*_ are the resonator frequency, the effective mass, and quality factor of the NR, respectively. The decay rate of the NR is *γ*_*n*_ = *ω*_*n*_/*Q*_*f*_ = 40 kHz, and the coupling strength is *g* = 0.06. For MFs, there are no experimental values for the lifetime *κ*_*M*_ of the MFs and the QD-MF coupling strength *β* in the recent literature. According to a few recent experimental reports^16^,^17^,^18^,^19^,^20^,^21^, it is reasonable to assume that the lifetime of the MFs is *κ*_*M*_ = 0.1 MHz. Since the QD-MF coupling strength is dependent on their distance, we expect *β* = 0.05 GHz via adjusting the distance between the hybrid QD-NR system and the iron chains.

**Figure 2:**
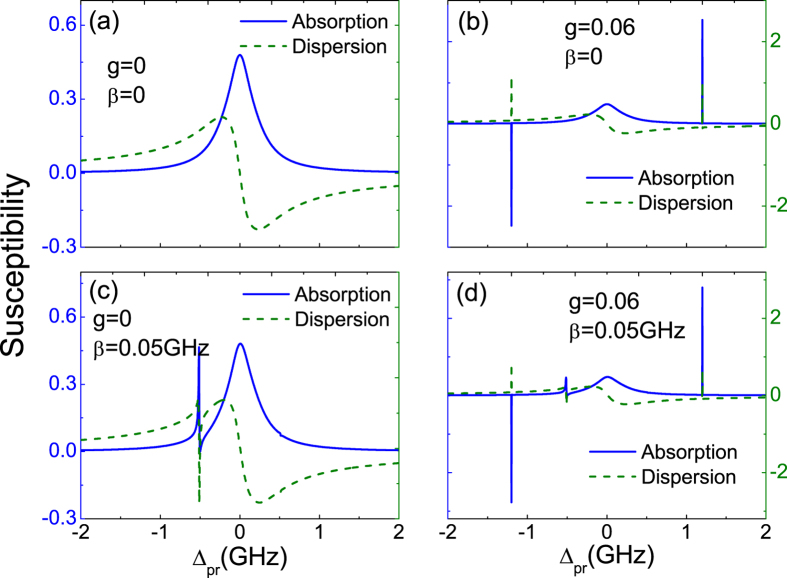
The absorption (the blue curve) and dispersion (the green curve) spectra of probe field as a function of the probe detuning Δ_*pr*_ under different conditions. (**a**) Without considering any coupling, i.e., *g* = 0 and *β* = 0. (**b**) The QD-NR coupling strength is *g* = 0.06 and *β* = 0. (**c**) The QD-MF coupling strength is *β* = 0.05 GHz and *g* = 0. (**d**) Considering both the QD-NR coupling and QD-MF coupling, i.e., *g* = 0.06 and *β* = 0.05 GHz. The parameters used are Γ_1_ = 0.3 GHz, Γ_2_ = 0.15 GHz, *γ*_*m*_ = 40 kHz, *ω*_*n*_ = 1.2 GHz, *κ*_*M*_ = 0.1 MHz, 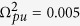
(GHz)^2^, Δ_*M*_ = −0.5 GHz, and Δ_*pu*_ = 0.

Figure 2(a) shows the absorption (*Imχ*^(1)^) and dispersion (*Reχ*^(1)^) properties of the QD as functions of probe-exciton detuning Δ_*pr*_ = *ω*_*pr*_ − *ω*_*e*_ at the detuning Δ_*pu*_ = 0 without considering any coupling (*g* = 0, *β* = 0), which indicates the normal absorption and dispersion of the QD, respectively. After turning on the QD-NR coupling (*g* = 0.06) and without considering the QD-MF coupling (*β* = 0), two sharp peaks appear in both the absorption and dispersion spectra as shown in Fig. 2(b). It is clear that the two sharp peaks at both sides of the spectra just correspond to the frequency of the NR. The physical origin of this result is due to mechanically induced coherent population oscillation, which makes quantum interference between the resonator and the beat of the two optical fields via the QD when the probe-pump detuning is equal to the NR frequency^45^. This reveals that if fixing the pump field on-resonance with the exciton and scanning through the frequency spectrum, the two sharp peaks can obtain immediately in the coherent optical spectra, which indicates a scheme to measure the frequency of the NR. This phenomenon stems from the quantum interference between the vibration of NR and the beat of the two optical fields via the exciton when probe-pump detuning *δ* is adjusted equal to the frequency of the NR. The QD-NR coupling play a key role in the hybrid system, and if we ignore the coupling (*g* = 0), the above phenomenon will disappear completely as shown in Fig. 2(a).

Compared with Fig. 2(b), in Fig. 2(c) we consider the QD-MF coupling without taking the QD-NR coupling into account, i.e. the condition of *g* = 0 and *β* = 0.05 GHz. As the MFs appear in the ends of iron chains and coupled to the QD, both the probe absorption (the blue curve) and dispersion (the green curve) spectra present an remarkable signature of MFs under Δ_*M*_ = −0.5 GHz. The physical origin of this result is due to the QD-MF coherent interaction and we can interpret this physical phenomenon with dressed state between the exciton and MFs. The QD coupled to the nearby MF *γ*_1_ will induce the upper level of the state 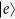 to split into 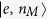 and 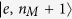 (*n*_*M*_ denotes the number states of the MFs). The left peak in the coherent optical spectra signifies the transition from 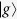 to 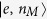 while the right peak is due to the transition of 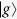 to 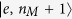
43. To determine the signals in Fig. 2(c) are the true MFs rather than other effect induced the like Majorana signatures, two queries should be clarified. The first one is the Kondo effect. The Kondo effect is usually associated with strong coupling to two normal leads in electrical detection scheme, and a superconducting gap is expected to suppress the effect when the gap is larger than the Kondo temperature. The experimental reports of Nadj-Perge group demonstrated that the disappearance of edge-localized zero-bias peaks when the underlying superconductivity is suppressed, which provides another evidence to prove MFs are associated with superconductivity and not with other phenomena such as the Kondo effect^21^. Therefore, in order to restrain the Kondo effect, the scheme of the chain of Fe atoms fabricated on top of a superconductor Pb substrate is one beneficial scheme to detect MFs. The second one is to distinguish the signature induced by the coupling between the normal electrons and the QD. We have introduced a tight binding Hamiltonian^46^ to describe the electrons in whole iron chains. To compare with the MFs signals, the parameters of normal electrons are chosen the same as MFs’ parameters. The numerical results indicate that there is no signal in the coherent optical spectra, which means that the signatures in the coherent optical spectra are the true MFs signals (not shown in the figures)^46^. Furthermore, if we consider both the two kinds coupling, i.e. the QD-NR coupling (*g* = 0.06) and QD-MFs coupling (*β* = 0.05 GHz) as shown in Fig. 2(d), not only the two sharp peaks locate at the NR frequency induced by its vibration, i.e. two peaks locate at Δ_*pr*_ = ±1.2 GHz (*ω*_*n*_ = 1.2 GHz), there is also MFs signal appear at Δ_*pr*_ = −0.5 GHz (Δ_*M*_ = −0.5 GHz) induced by the QD-MF coupling.

**Figure 3:**
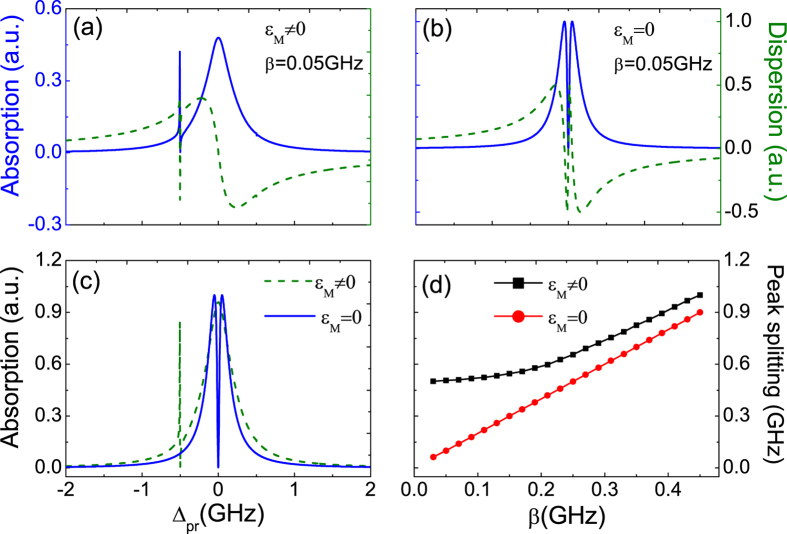
(**a,b**) show the probe absorption (the blue curve) and dispersion (the green curve) spectra with QD-MF coupling strengths *β* = 0.05 GHz under 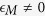 and 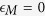, respectively. (**c**) The probe absorption spectrum under 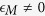 (the green curve) and 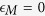 (the blue curve), respectively. (**d**) The linear relationship between the distance of peak splitting and the coupling strength of QD-MF *β*.

In Fig. 2(c), we only consider the situation of 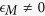. Actually, if the iron chains length *l* is much larger than the Pb superconducting coherent length *ξ*, 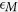 will approach zero. It is necessary to consider the conditions of 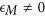 and 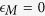, and we define them as coupled MFs mode (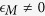) and uncoupled MFs mode (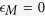), respectively. Figure 3(a,b) show the absorption and dispersion spectra as a function of detuning Δ_*pr*_ with QD-MF coupling constants *β* = 0.05 GHz under 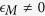 and 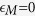, respectively. Compared with the coupled MFs mode, the uncoupled QD-MF Hamiltonian will reduce to *H*_*MF*−*QD*_ = *iħβ*(*S*^−^*f*^†^ − *S*^+^*f*) which is analogous J-C Hamiltonian of standard model under 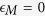, and the probe absorption spectrum (the blue curve) shows a symmetric splitting as the QD-MF coupling strength *β* = 0.05 GHz which is different from of coupled MFs mode presenting unsymmetric splitting at detuning Δ_*M*_ = −0.5 GHz. Our results therefore reveal that the signals in the coherent optical spectra are real signature of MFs, and the optical detection scheme can work at both the coupled Majorana edge states and the uncoupled Majorana edge states.

In Fig. 3(c), we further make a comparison of the probe absorption spectrum under the coupled MFs mode (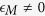) and uncoupled MFs mode (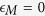). It is obvious that the probe absorption spectrum display the analogous phenomenon of electromagnetically induced transparency (EIT)^47^ under both the two conditions. The dip in the probe absorption spectrum goes to zero at Δ_*pr*_ = 0 and Δ_*pr*_ = −0.5 GHz with 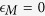 and 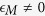, respectively, which means the input probe field is transmitted to the coupled system without any absorption. Such a phenomenon is attributed to the destructive quantum interference effect between the Majorana modes and the beat of the two optical fields via the QD. Once the beat frequency of two lasers *δ* is close to the resonance frequency of MFs, the Majorana mode starts to oscillate coherently resulting in Stokes-like (Δ_*S*_ = *ω*_*pu*_ − *ω*_*M*_) and anti-Stokes-like (Δ_*AS*_ = *ω*_*pu*_ + *ω*_*M*_) scattering of light from the QD. The Stokes-like scattering is strongly suppressed due to highly off-resonant with the exciton frequency, while the anti-Stokes-like field interfere with the near-resonant probe field and modify the probe field spectrum. The Majorana modes play a vital role in the coupled system, and we refer the phenomenon as Majorana modes induced transparency (MMIT).

On the other hand, we propose a means to determine the QD-MF coupling strength *β* via measuring the distance of the two peaks with increasing the QD-MF coupling strength in the probe absorption spectrum. Figure 3(d) indicates the peak-splitting width as a function of *β* under the condition of the coupled MFs mode (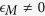) and the uncoupled MFs mode (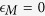) which follows a nearly linear relationship. It is obvious that the two lines have a slight deviation. With increasing the coupling strength, the deviation becomes slighter. It is essential to enhance the coupling strength for a clear peak splitting via adjusting the distance between the QD and the nearby MFs. In this case the coupling strength can obtain immediately via directly measuring the distance of the two peaks from the probe absorption spectrum.

**Figure 4:**
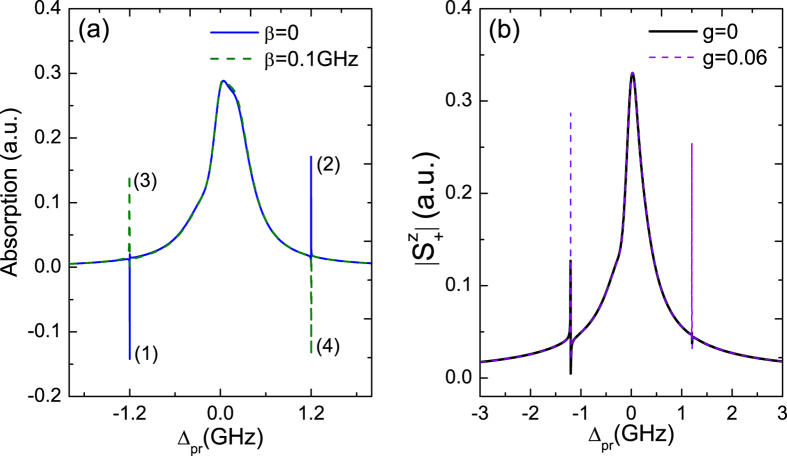
(**a**) The probe absorption spectrum as a function of the probe detuning Δ_*pr*_ with considering (the blue curve, *β* = 0.1 GHz) and without considering (the green curve, *β* = 0) the QD-MF coupling under the QD-NR coupling strength *g* = 0.06. (**b**) The exciton resonance spectrum as a function of Δ_*pr*_ with *g* = 0 and *g* = 0.06 at the QD-MF coupling strength *β* = 0.1 GHz. Δ_*M*_ = −1.2 GHz, Δ_*pu*_ = 0.05 GHz, 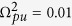
(GHz)^2^, and the other parameters used are the same as Fig. 2.

As shown in Fig. 2(d), there are not only two sharp peaks locate at the NR frequency induced by its vibration but also the MFs signal appear at Δ_*pr*_ = Δ_*M*_ induced by the QD-MF coupling in the probe absorption spectrum (the blue curve) under the two kinds coupling. In Fig. 4(a), we further consider switching the detuning Δ_*M*_ = −0.5 GHz to Δ_*M*_ = −1.2 GHz at small exciton-pump detuning Δ_*pu*_ = 0.05 GHz. It is obvious that the resonance amplification process (1) and the resonance absorption process (2) in the probe absorption spectrum without considering the QD-MF coupling (the blue curve, *β* = 0) will accordingly transform into the the resonance absorption process (3) and the resonance amplification process (4) due to the QD-MF coupling (the green curve, *β* = 0.1 GHz). Return to Fig. 1(a), there are two kinds of coupling which are QD-NR coupling and QD-MF coupling in the hybrid system. For the QD-NR system, the two sharp peaks in the probe absorption corresponding to the resonance amplification (1) and absorption process (2) can be elaborated with dressed states 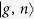, 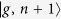, 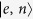, 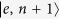 (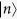 denotes the number state of the resonance mode), and the two sharp peaks indicate the transition between the dressed states^45^. However, once MFs appear in the ends of iron chains and coupled to the QD, the ground state 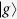 and the exciton state 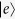 of the QD will also modify by the number states of the MFs *n*_*M*_ and induce the Majorana dressed states 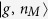, 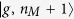, 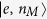, 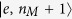. With increasing the QD-MF coupling, the Majorana dressed states will affect the amplification (1) and absorption process (2) significantly, and even realize the inversion between the absorption (3) and amplification (4) process due to the QD-MF coherent interaction (the green curve).

In Fig. 4(b), we introduce the exciton resonance spectrum to investigate the role of NR in the coupled QD-NR, which is benefited for readout the exciton states of QD. Adjust the detuning Δ_*M*_ = −0.5 GHz to Δ_*M*_ = −1.2 GHz, the location of the two sideband peaks induced by the QD-MF coupling coincides with the two sharp peaks induced by the vibration of NR, and the NR is resonant with the coupled QD-MF system making the coherent interaction of QD-MF more strong. Figure 4(b) shows the exciton resonance spectrum of the probe field as a function of the probe detuning Δ_*pr*_ with the detuning Δ_*pu*_ = 0.05 GHz under the coupled MFs mode 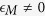. The black and purple curves correspond to *g* = 0 and *g* = 0.06 for the QD-MF coupling *β* = 0.1 GHz, respectively. It is obvious that the role of NR is to narrow and to increase the exciton resonance spectrum. The NR therefore behaves as a phonon cavity will enhance the sensitivity for detecting MFs.

## Discussion

We have proposed an all-optical means to detect the existence of MFs in iron chains on the superconducting Pb surface with a hybrid QD-NR system. The signals in the coherent optical spectra indicate the possible Majorana signature, which provides another supplement for detecting MFs. Due to the vibration of NR, the exciton resonance spectrum becomes much more significant and then enhances the detection sensitivity of MFs. In addition, the QD-NR coupling in our system is a little feeble, while ref. 48 presents a strong QD-NR coupling and the coupling strength can reach kilohertz, which is beneficial for MFs detection. On the other hand, if we consider embedding a metal nanoparticle-quantum dot (MNP-QD) complex^43^,^45^ in the NR, the surface plasmon induced by the MNP will enhance the coherent optical property of QD, which may be robust for probing MFs. The concept proposed here, based on the quantum interference between the NR and the beat of the two optical fields, is the first all-optical means to probe MFs. This coupled system will provide a platform for applications in all-optically controlled topological quantum computing based on MFs.

## Methods

The Heisenberg operators are rewritten as the sum of its steady-state mean value and a small fluctuation with zero mean value: 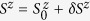, *S*^−^ = *S*_0_ + *δS*^−^, *f* = *f*_0_ + *δf*, and *Q* = *Q*_0_ + *δQ*. Inseting the operators into Eqs (5)–(8), we abtain


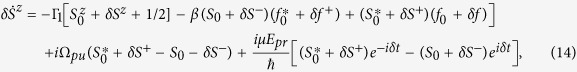



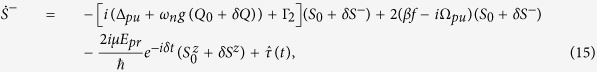



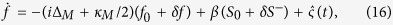



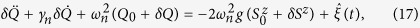


Solving them we obtain two equation sets. The steady-state mean equation set are


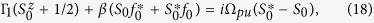



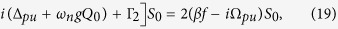



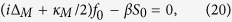



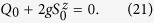


The solutions of the above equation set determine the population inversion as shown in Eq. (11). The fluctuation equation set are


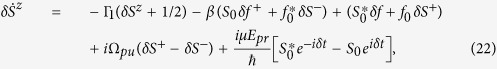



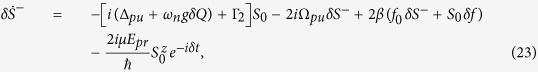



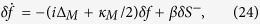



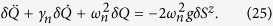


Solving the above equation set, we obtain Eq.(12) and Eq. (13).

## Additional Information

**How to cite this article**: Chen, H.-J. *et al*. Robust signatures detection of Majorana fermions in superconducting iron chains. *Sci. Rep.* **6**, 36600; doi: 10.1038/srep36600 (2016).

**Publisher’s note**: Springer Nature remains neutral with regard to jurisdictional claims in published maps and institutional affiliations.
